# Surgical treatment of giant seminal vesicle cyst with ureteral compression: a case report

**DOI:** 10.3389/fsurg.2025.1503368

**Published:** 2025-03-03

**Authors:** Zhuoran Gu, Liang Sun, Wentao Zhang, Jiang Geng, Lei Jiang, Yifan Chen

**Affiliations:** ^1^ Department of Urology, Shanghai Tenth People’s Hospital, School of Medicine, Tongji University, Shanghai, China; ^2^ Urologic Cancer Institute, School of Medicine, Tongji University, Shanghai, China; ^3^ Department of Urology, Bengbu First People’s Hospital, Anhui, China

**Keywords:** seminal vesicle cyst, ureteral compression, hematuria, laparoscopic surgery, ureteric stent insertion

## Abstract

**Background:**

Seminal vesicle cysts (SVCs) are rare benign diseases in men and are commonly asymptomatic. Giant SVCs with complications there are no standard treatments for SVCs, however surgical intervention is required for giant SVCs accompanied with complications.

**Case summary:**

We present one case of a 49-year-old male patient diagnosed with giant SVC. Chief complaint of this patient was persistent gross hematuria for 1 week. Both a computed tomography (CT) and magnetic resonance imaging (MRI) scans indicated the presence of a cystic mass in left seminal vesicle with hemorrhage, of which the maximum diameter is 6.5 cm. Additionally, Giant SVC squeezed the prostate and lower ureter, leading to the dilatation of the left upper ureter and hydronephrosis. After a thorough preoperative evaluation, a laparoscopic resection of SVC and left ureteral stenting were performed. The subsequent pathological analysis identified a seminal vesicle cyst inflammatory infiltration. Postoperative follow-up indicated no abnormalities in left seminal vesicle.

**Conclusion:**

Laparoscopic surgery is recommended for giant SVCs with complications.

## Introduction

The seminal vesicle cyst (SVC) is a rare benign condition in men, with an incidence of approximately 0.005% (1). SVC is typically small and characterized by bladder irritation or perineal pain, usually without additional complications. The giant SVC, however, may be associated with hemorrhage or infection, though ureteral compression is extremely uncommon (2). We report a case of a 49-year-old male initially admitted to the hospital with gross hematuria. An ultrasound of the urinary system revealed dilation of the upper left ureter and separation of the left renal pelvis and calyces. Additionally, an uneven hypoechoic mass was observed in the left seminal vesicle. Subsequent abdominal and pelvic CT and MRI scans confirmed a large cystic mass in the left seminal vesicle, accompanied by hemorrhage and compression of the lower left ureter. The patient subsequently underwent 3D laparoscopic resection of the left seminal vesicle cyst with left ureteral stenting. Postoperative pathology confirmed the diagnosis of a left giant SVC.

## Case presentation

**Figure 1:**
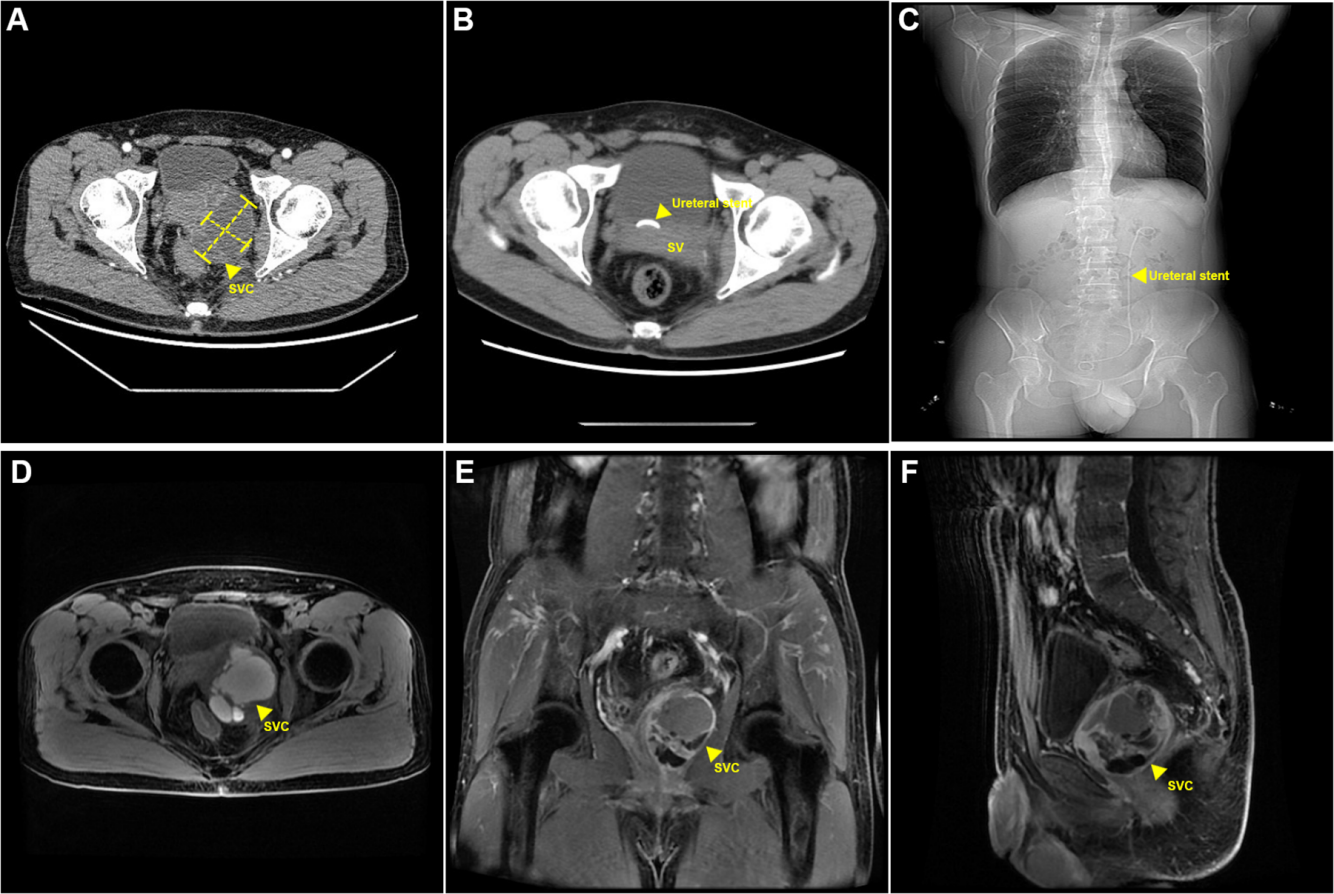
Perioperative radiographic findings. **(A)** Preoperative CT findings: cystic lesion in the left seminal vesicle. **(B,C)** Postoperative CT findings: postoperative left ureteral stent placement. **(D–F)** Postoperative MRI findings: Cystic lesion in the left seminal vesicle with hemorrhage. SVC, seminal vesical cyst.

One 49-year-old male patient was admitted to the hospital with hematuria. Digital rectal examination (DRE) indicated a Grade I enlarged prostate, firm in texture, without tenderness. The central sulcus was shallow, and a cystic mass approximately 5.0 cm in diameter, soft in texture, and with poorly defined borders, was palpable on the left side of the prostate. No blood was observed on the examination glove following the DRE. Ultrasound examination showed left ureteral dilation accompanied by left-sided hydronephrosis and a cystic mass in the left seminal vesicle. A further CT urography (CTU) of the urinary system revealed a 6.0 cm × 5.0 cm cystic, low-density lesion in the left prostate-seminal vesicle region with septations, which enhanced after contrast administration. The prostate was displaced by the lesion (Figure 1A). An MRI of the pelvis confirmed a 6.5 cm × 5.5 cm cystic mass in the left seminal vesicle, with a capsule surrounding the mass and multiple internal septations with fluid levels. The left seminal vesicle was compressed and displaced. T1-weighted and T2-weighted images showed high signal intensity, while diffusion-weighted imaging (DWI) showed mildly high signal intensity. Contrast-enhanced imaging revealed enhancement of the cyst wall (Figure 1D–F). In addition, since the onset of the condition, the patient remained conscious and mentally stable, with normal bowel habits and no significant weight changes. The patient had no history of prior surgeries, trauma, or psychiatric disorders. Additionally, there was no family history of similar conditions or other significant medical diseases. The patient did not smoke or consume alcohol. Based on the patient's clinical symptoms and imaging findings, a preliminary diagnosis of a large hemorrhagic seminal vesicle cyst with left ureteral compression was made.

**Figure 2:**
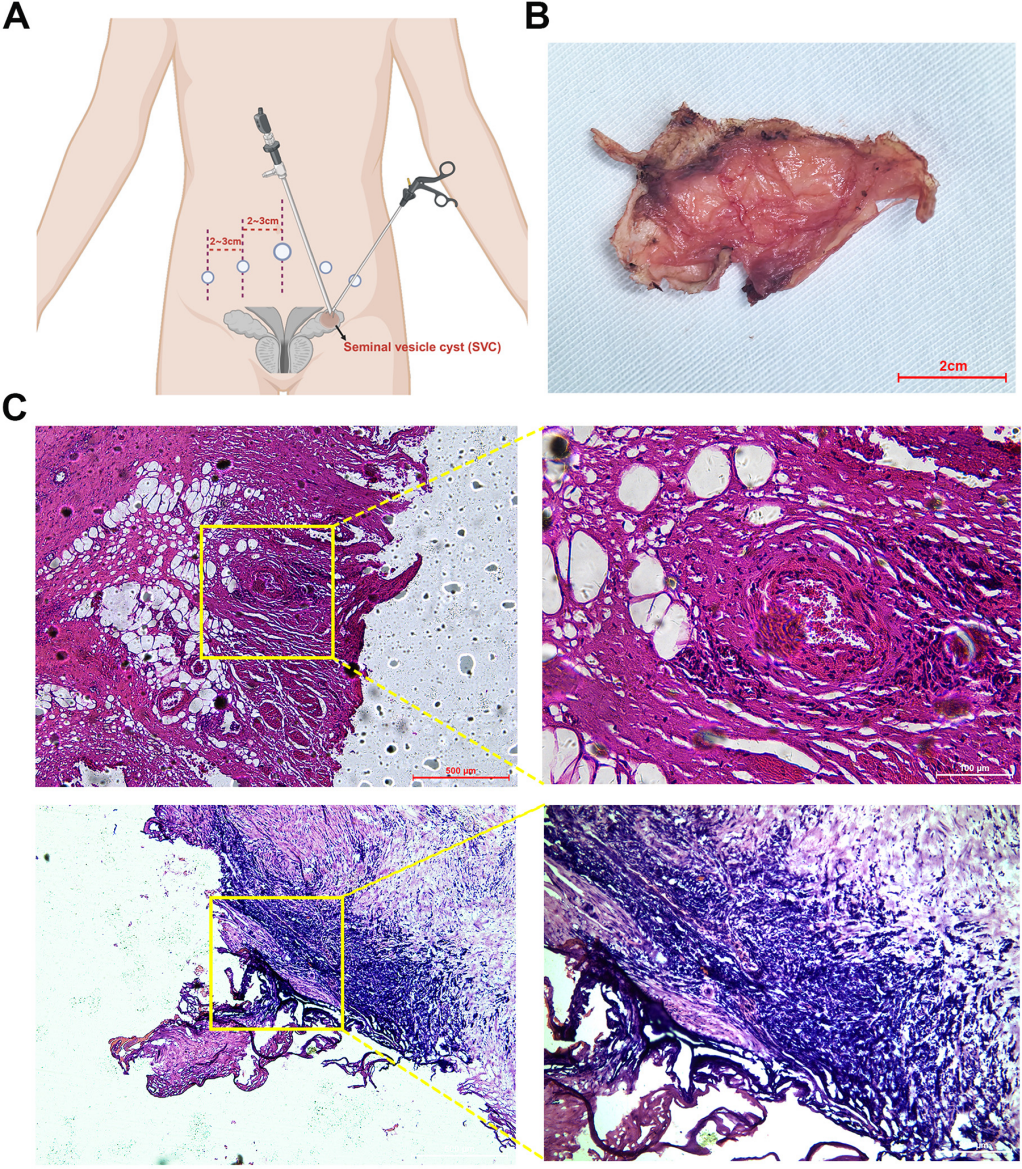
Postoperative specimen and histological findings. **(A)** Schematic diagram of laparoscopic port placements. **(B)** Excised seminal vesicle cyst specimen. **(C)** Histological image of the seminal vesicle cyst stained with hematoxylin and eosin (HE).

The patient underwent 3D laparoscopic resection of the left seminal vesicle cyst and placement of a left ureteral stent. The surgical procedure and intraoperative findings were as follows: the patient was positioned in the lithotomy position, a ureteroscope was inserted through the urethra, revealing a mound-shaped verumontanum. Attempts to pass a guidewire into the verumontanum were unsuccessful. The urethral mucosa appeared smooth and normal. The scope was advanced into the bladder, where the bladder mucosa was smooth and both ureteral orifices had a fish-mouth appearance. A zebra guidewire was inserted into the left ureter, and under its guidance, a 6F ureteral stent was placed. The ureteroscope was withdrawn, and a Foley catheter was inserted. The patient was repositioned to a supine position with a 25-degree Trendelenburg tilt. Five ports were utilized during the surgical procedure (Figure 2A). After disinfecting the surgical field, a 1.5 cm incision was made below the umbilicus. The abdominal wall was elevated, and the layers were incised to access the abdominal cavity. A 10 mm trocar was inserted, and the incision was closed to prevent air leakage. Carbon dioxide was insufflated to maintain a pneumoperitoneum at 14 mmHg. A 3D laparoscope was introduced, and four additional trocars were placed under direct visualization at the following locations: along the lateral margins of the left and right rectus abdominis muscles, and 2 cm lateral to these trocars, where 5 and 12 mm trocars were inserted. The left peritoneum was opened, revealing a mass filling the left pelvis, adherent to the surrounding tissues. The bladder was pushed to the contralateral side by the mass. Sharp and blunt dissection was used to free the mass from surrounding tissues. The left ureter was identified superior to the mass, freed, and retracted laterally with a rubber band to expose the mass further. Dissection of the mass was continued posteriorly, separating it from the rectum. After fully exposing the mass, an incision was made along its edge, and it was gradually dissected deeper until the mass was excised from the pelvic cavity. Once the surgical specimen was removed from the body, the tumor was incised, releasing a large amount of brown fluid. A tissue sample was sent for frozen pathology, which suggested a benign lesion with inflammatory reaction and a few glandular-like structures. After injecting 200 ml of saline into the bladder, no leakage was observed. The surgical area was inspected for active bleeding, none was found, and a latex drainage tube was placed. The peritoneum was closed with continuous sutures using 3-0 absorbable sutures. The incision was closed in layers.

The estimated blood loss was approximately 50 ml, and the surgery lasted about 2 h. Postoperative pathology confirmed a seminal vesicle cyst (5.0 cm × 3.5 cm × 3.0 cm) with cyst wall fibrosis, collagenization, and marked inflammatory response. Some areas showed non-atypical glandular/ductal structures consistent with seminal vesicle gland tissue. Immunohistochemical results indicated that 34βE12, CK8/18, and CK7 were positive (+); while CK20, PSA, and P504S were negative (−). At the 1-month follow-up, the patient's gross hematuria had resolved, and the left ureteral dilation and hydronephrosis had disappeared (Figure 1B,C).

## Discussion

Seminal vesicle cysts (SVCs) are categorized as either congenital or acquired. Congenital SVCs typically result from developmental abnormalities of the mesonephric duct during the 4th to 13th weeks of gestation (3). During this period, the mesonephric duct forms the ureter, renal pelvis, calyces, and evolves into the epididymal duct, vas deferens, seminal vesicles, and ejaculatory ducts. Malformations in this developmental window can lead to ejaculatory duct atresia, seminal vesicle obstruction, and cyst formation. These may occur alongside other urogenital malformations, such as ipsilateral renal dysplasia or agenesis, ureteral dysplasia, or ectopic ureteral openings into the seminal vesicle (3). When congenital SVC is associated with ipsilateral renal agenesis and ectopic ureteral openings into the seminal vesicle, it is referred to as Zinner's syndrome (4). Acquired SVCs are often associated with infections of the genitourinary system, such as prostatitis, seminal vesiculitis, or ejaculatory duct obstruction following prostate surgery (5). The differential diagnosis for SVCs includes Müllerian duct cysts, prostatic utricle cysts, epididymal cysts, hydatid cysts, and benign or malignant neoplasms of the seminal vesicle, such as adenocarcinomas or papillary adenomas. Infections resulting in pelvic abscesses may also mimic cystic lesions but are often accompanied by systemic symptoms. Zinner's syndrome presents a characteristic triad of ipsilateral renal agenesis, ectopic ureter, and SVCs, making it a critical entity in the differential diagnosis.

The size of the SVC often correlates with clinical symptoms. SVCs smaller than 5.0 cm are typically asymptomatic, while those 5.0 cm or larger are more likely to cause noticeable symptoms. Digital rectal examination (DRE) is a simple, effective diagnostic tool with an accuracy rate of up to 79%. It may reveal a cystic mass in the seminal vesicle region posterior to the prostate, sometimes associated with other urogenital malformations such as hypospadias or cryptorchidism. Imaging is crucial for diagnosis, with ultrasound being the preferred initial screening method due to its simplicity, low cost, and high sensitivity and specificity for SVC (6). Ultrasonography typically reveals a cystic lesion between the posterior bladder wall and the base of the prostate, lateral to the midline. When complicated by hemorrhage or infection, the cystic lesion may appear heterogeneous. Transrectal ultrasound provides a clearer view of the seminal vesicles. Further assessment with abdominal ultrasound and high-frequency probes can confirm associated anomalies such as renal agenesis or cryptorchidism. CT or MRI can assess the cyst's size, location, and nature, aiding in preoperative planning (4). These modalities allow for precise localization and differential diagnosis based on the cyst's morphology, density, signal characteristics, and relationship with adjacent organs.

SVCs are generally not associated with complications, although large SVCs can be complicated by hemorrhage or infection. Cases of large SVCs causing hydronephrosis due to ureteral compression are extremely rare (2). To our knowledge, this case represents the only reported instance. The patient, a 49-year-old male, presented with hematuria. Ultrasound revealed a large cystic lesion in the left seminal vesicle, accompanied by left ureteral dilation and hydronephrosis, without evidence of renal agenesis. CT and MRI confirmed a large left seminal vesicle cyst with hemorrhage, compressing the ureter and causing significant hydronephrosis. The preoperative diagnosis was an acquired SVC.

In clinical practice, patients with small SVC and no significant symptoms are typically managed conservatively with close follow-up. Indications for SVC surgery include the presence of clinical symptoms or ejaculatory duct obstruction resulting in infertility. Surgical options include transurethral cyst unroofing, transrectal aspiration, laparoscopic cyst excision, and open surgery (7). Due to the high recurrence rate following cyst aspiration and unroofing, these procedures are recommended only for elderly patients, those with significant comorbidities, or individuals who cannot tolerate more invasive procedures. They are unsuitable for younger patients or those with fertility concerns. Conventional open surgical approaches, including transabdominal, transperineal, transvesical, and transcoccygeal routes, pose significant challenges due to the deep pelvic location of the seminal vesicles. These challenges include limited surgical exposure, extensive trauma, prolonged operative time, and increased blood loss. Given the higher risk of intraoperative bleeding, greater tissue trauma, and increased postoperative complications, open surgery is no longer considered the preferred method for managing seminal vesicle cysts. Laparoscopic surgery offers significant advantages, including minimal invasiveness, reduced bleeding, faster recovery, and fewer complications, making it the preferred approach among many surgeons (8, 9). The *Da Vinci* robotic surgical system facilitates precise dissection and visualization of seminal vesicle cysts and adjacent anatomical structures, enabling accurate identification and assessment of the ejaculatory duct anatomy and potential variations. It offers distinct advantages in cyst excision, hemostasis, and localized suturing. Additionally, robot-assisted laparoscopic surgery allows for the complete resection of the SVC, followed by meticulous suturing and closure of the residual cystic structure, thereby restoring the seminal vesicle to its physiological volume and supports effective reconstruction of the ejaculatory duct (10). Some studies have also reported favorable outcomes with transurethral incision of the SVC combined with electrocautery of the seminal vesicle wall (11). In this case, after completing preoperative evaluations, the patient underwent laparoscopic surgery with left ureteral stent placement. Inappropriate utility of laparoscopic energy application can lead to thermal injury, which may result in ureteric strictures. To minimize this risk, we ensured the use of precise and controlled energy settings during the procedure. Additionally, the placement of a ureteric stent served as a tactile guide to help identify the ureter and avoid direct or collateral energy application. By employing these precautions, the risk of ureteric strictures due to thermal injury was effectively mitigated in this case. The surgery was associated with minimal bleeding and a clear surgical field, and postoperative pathology confirmed the diagnosis of an acquired SVC. The patient's symptoms resolved postoperatively, with no cyst recurrence observed on follow-up, and the prognosis was favorable.

## Conclusion

To prevent compression of surrounding tissues and subsequent complications, timely surgical intervention is required for the giant SVC, with laparoscopic surgery being the recommended approach.

## Data Availability

The original contributions presented in the study are included in the article/Supplementary Material, further inquiries can be directed to the corresponding authors.
